# A Lattice-Hinge-Design-Based Stretchable Textile Microstrip Patch Antenna for Wireless Strain Sensing at 2.45 GHz

**DOI:** 10.3390/s23218946

**Published:** 2023-11-03

**Authors:** Abdul Wahab Memon, Benny Malengier, Patrick Van Torre, Lieva Van Langenhove

**Affiliations:** 1 Centre of Textile Science and Engineering, Department of Materials, Textiles and Chemical Engineering, Ghent University, 9052 Ghent, Belgium; benny.malengier@ugent.be (B.M.); lieva.vanlangenhove@ugent.be (L.V.L.); 2 Department of Textile Engineering, Mehran University of Engineering & Technology, Jamshoro 76020, Pakistan; 3 Department of Information Technology, Faculty of Engineering and Architecture Imec-IDLab, Ghent University, 9052 Ghent, Belgium; patrick.vantorre@ugent.be

**Keywords:** wearable textile antenna, multifunctional antenna, lattice hinge design, e-textile, polydimethylsiloxane, stretchable antenna, strain sensor

## Abstract

The manuscript presents a novel approach to designing and fabricating a stretchable patch antenna designed for strain sensing and the wireless communication of sensing data at the same time. The challenge lies in combining flexible and stretchable textile materials with different physical morphologies, which can hinder the adhesion among multiple layers when stacked up, resisting the overall stretchability of the antenna. The proposed antenna design overcomes this challenge by incorporating a lattice hinge pattern into the non-stretchable conductive e-textile, transforming it into a stretchable structure. The innovative design includes longitudinal cuts inserted in both the patch and the ground plane of the antenna, allowing it to stretch along in the perpendicular direction. Implementing the lattice hinge pattern over the conductive layers of the proposed patch antenna, in combination with a 2 mm thick Polydimethylsiloxane (PDMS) substrate, achieves a maximum of 25% stretchability compared to its counterpart antenna without a lattice hinge design. The stretchable textile antenna resonates around a frequency of 2.45 GHz and exhibits a linear resonant frequency shift when strained up to 25%. This characteristic makes it suitable for use as a strain sensor. Additionally, the lattice hinge design enhances the conformability and flexibility of the antenna compared to that of a solid patch antenna. The realized antenna gains in the E and H-plane are measured as 2.21 dBi and 2.34 dBi, respectively. Overall, the presented design offers a simple and effective solution for fabricating a stretchable textile patch antenna for normal use or as a sensing element, opening up possibilities for applications in the communication and sensing fields.

## 1. Introduction

Recent adaptations of smart systems for on-body wearable applications for human well-being, health monitoring, and observing human movements have grabbed attention [1,2]. Following recent developments, more flexible electronic components are becoming a part of wearable systems as they offer greater flexibility, comfort during wearability, and ease of handling [3,4]. Wearable sensors, energy-harvesting devices, epidermal electronics, and a variety of electronic circuits have been developed and used in health monitoring [3,5,6,7]. To convey sensory information and access remote diagnosis through wearable wireless communication, an antenna is required. Real-time measurements related to physiological aspects such as heart rate, human body movements, intrinsic strain measurements, environmental humidity, and body temperature through wireless sensor networks (WSNs) and body sensor networks (BSNs) require more flexible electronic components [8,9,10,11]. Applications such as humanoid robotics, human–machine interactions, and prosthetics also require flexible electronic components. WSNs and BSNs integrated with functional devices for such end-use applications demand flexibility, stretchability, and conformability from embedded devices in these networks. Materials that offer such a variety of properties create the possibility of making flexible electronic components [12]. Additionally, such materials can also be used to produce multifunctional devices with added functions. Many flexible antennas have been fabricated and are effective for wearable applications, but they are limited to a single end-use application, namely the receiving or transmission of electromagnetic (EM) radiations [13,14]. In some other reported research, an antenna and a strain sensor are typically treated as separate devices within a wearable system [15,16]. Applications, such as strain sensing parallel to communication, require antennas to be made of stretchable materials with a high modulus that can attain a high elongation before break. Antenna sensitivity toward stretching can be utilized as a strain-sensing factor [17]. During practical end-use applications, when a stretchable antenna experiences uniaxial strain or deformation, its resonance frequency changes accordingly. This change in antenna resonance frequency can serve as a signature of deformation due to applied strain, indicating the potential use of a stretchable antenna as a strain sensor [17,18,19,20]. Using a multifunctional antenna in a wearable system can be beneficial in reducing the number of devices in the wearable system without compromising its functionality.

**Figure 1:**
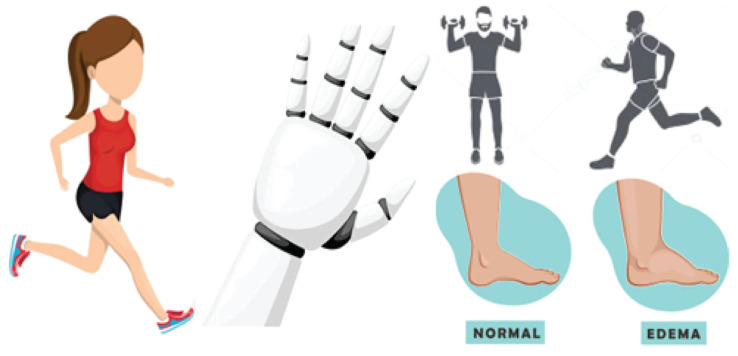
Overview of the potential strain-sensing end-use applications for multifunctional stretchable textile antennas.

The use of multifunctional devices also minimizes the problem of complex wiring, which is prone to damage and is required to acquire sensory data in wearable smart systems. Similarly, a wearable system with multifunctional devices can solve integration challenges with any garment [4,21]. Considering these practical end-use challenges and limitations, a wearable system with multifunctional devices that offers equal functionalities, similar to a wearable system with more nodes and sensors required for separate functions to analyze but with less complexity, would be advantageous [22]. Figure 1 illustrates an overview of the potential end-use applications of a strain sensor stretchable antenna, including the detection of Edema disease, monitoring the joint angles in robotic hands to control movement, assessing inflated food packages due to spoilage, and various fields such as sports, healthcare, rehabilitation, structural health monitoring, and smart packaging. These are some of the possible applications where the proposed stretchable textile microstrip patch antenna can be used.

This manuscript presents a fully textile-based, flexible, and stretchable antenna. Our proposed work focuses on fabricating a stretchable textile patch antenna for strain sensing and communication, which can be used in wearable applications. Furthermore, we present a novel and simpler method for fabricating a stretchable antenna on a non-stretchable e-textile fabric. Our research follows an adapted and simplified antenna design, making it an interesting progression. The antenna is realized using a non-stretchable turned stretchable e-textile fabric, accompanied by a transparent, flexible, and stretchable non-conductive polydimethylsiloxane (PDMS) substrate.

This paper is organized as follows: Section 2 presents the state-of-the-art stretchable antennas and the general strategy adopted in this research work to realize the stretchable textile microstrip patch antenna. Section 3 includes the materials and methods used to develop the stretchable textile microstrip patch antenna. The antenna design, its fabrication method, modeling, and simulation are described in Section 4. The methodology for characterizing the EM performance of the stretchable antenna is discussed in Section 5. The simulated and measured EM results of the developed antenna are discussed in detail in Section 6, and last, the key conclusions are discussed in Section 7.

## 2. State-of-the-Art Stretchable Antennas

In recent years, several stretchable antennas have been developed [13,23,24]. The choice to fabricate a stretchable antenna with the desired EM properties for a wearable system depends on various factors. These include the availability of stretchable materials, both conductive and non-conductive, the space available for antenna integration into the wearable system, and the required operating frequency range [25,26]. More importantly, the incompatibility of the physical parameters among the different materials used in fabricating a patch antenna makes the task of pasting multiple layers, while still leaving their stretchability intact, challenging.

Various conductive materials have been employed to construct stretchable antennas, including Silver-Polydimethylsiloxane (Ag-PDMS) composites, metal nanowires, and liquid metal alloys. However, the poor conductivity of these materials remains a significant concern [23,27,28]. While these materials offer improved stretchability, their EM performance in radio-frequency (RF) wireless technologies still requires enhancement. To address this issue, more recently [17], conductive textile fabrics such as copper and silver-coated polymeric textile fabrics have been utilized and given a serpentine structure. Consequently, using a highly conductive textile fabric with a serpentine structure provides better stretchability and suitable EM performance.

To fabricate a stretchable patch antenna, one option is to use conductive and dielectric materials that have a stretchable character. However, this way of constructing a stretchable patch antenna is difficult, as neither the conductive and dielectric materials will not be compatible in their stretchability. One will offer more stretchability than the other when sandwiched together in a patch antenna shape. Alternatively, the antenna design itself can be modified to allow for an extra amount of stretching, or a combination of both approaches can be employed. Each method of fabricating a stretchable antenna has its advantages and limitations. In recent developments, in an example where an antenna design was modified for a patch antenna to obtain stretchability, optimally patterned 3D structures such as serpentine and helical coils made from conventional non-stretchable materials like copper foil were utilized for fabricating stretchable patch antennas [12,17]. These structures were then deposited onto stretchable dielectric materials such as Ecoflex, Solaris, and PDMS. This approach involved a complex process of designing the antenna with a 3D serpentine pattern, simulating it, using specialized software to design and cut the intricate pattern, and finally, employing a delicate fabrication process to deposit the 3D serpentine patterns onto the stretchable material. Various parameters, including the arc angle, need to be considered while simulating serpentine-structure-based antennas [17,29]. The resulting serpentine patch antennas exhibited an improved stretchability, but they also resulted in a higher Surface Absorption Rate (SAR) due to the serpentine-structured ground plane. The limited surface area of the conductive material in the serpentine ground plane compared to a solid layer in the traditional patch antenna topology contributed to this increase in the SAR [30,31].

Other recent advancements in the field of stretchable antennas include the use of serpentine structures (stretchable three-dimensional structures with repeated unit cells of the same shape), materials such as silver nanowires (AgNWs), and liquid metal alloys [15,26,32,33,34]. In another notable development [17], for fabricating a stretchable antenna, the antenna was based on a serpentine structure. Here, a stretchable, knitted, silver-plated textile fabric (MedTex, Smethwick, UK) was converted into a serpentine structure first and then joined with the stretchable Ecoflex substrate to fabricate a stretchable patch antenna. Though the serpentine structure exhibited a high sensitivity toward stretching due to the use of stretchable conductive fabric over a stretchable dielectric substrate, the fabrication process in shaping the serpentine structure involved a few steps followed by a delicate step of depositing the serpentine conductive layers over the stretchable Ecoflex layer to ensure their integrity and shape remained intact. There is always a trade-off between the mechanical stretchability and EM performance of the meshed/serpentine antenna. Another recently reported approach [35] employed conventional non-stretchable electrodes or sheets (such as stiff metals like copper foil) patterned in a repeated unit cell to make them stretchable. These serpentine layers were combined with a PDMS substrate to fabricate a stretchable antenna. Other research has explored strategies such as Kirigami-like structures and twisted helical spring structures to enable stretchability in materials that were originally non-stretchable [36]. In terms of quantifying stretchability, AgNWs achieve a minimum of 15% stretchability, while serpentine-based antennas can achieve a maximum stretchability of 100% [17,26].

**Figure 2:**
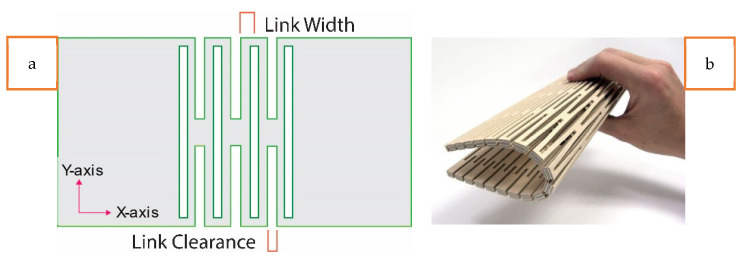
(**a**) The lattice hinge with parameters and (**b**) the lattice hinge pattern on a wooden sheet.

**Figure 3:**
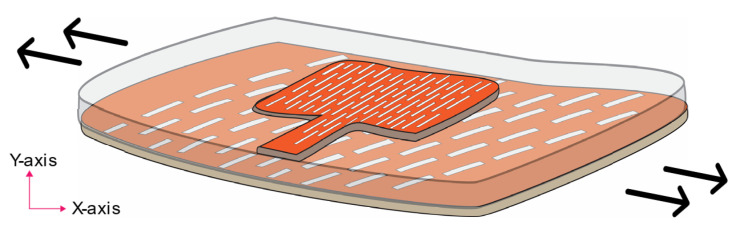
A schematic view of the LHD-based stretchable microstrip patch antenna.

Our research proposes a simpler, easy, and novel method for fabricating stretchable textile-based antennas, based on transforming plain conductive layers of a patch antenna into lattice hinge design (LHD) conductive layers. The structure of an LHD and its design parameters are shown in Figure 2a and Figure 2b, respectively. The novel LHD approach, also called living-hinge, is an innovative way of transforming non-stretchable materials into stretchable ones by introducing a lattice framework with uniquely shaped hinges and is adapted in our research work. The basic idea behind a lattice-hinge technique is to construct a framework of regularly shaped hinges throughout the surface of the non-stretchable material that helps the material to flex and expand while maintaining its structural integrity. Systematically positioning the lattice-hinge framework over the materials’ surface ensures their uniform and controlled stretchability. This design enables the transformation of a non-stretchable sheet (such as a wood sheet) into a stretchable and bendable one by patterning the sheet in a way that allows for deformation when stretched or subjected to uniaxial strain. Unlike serpentine structures, this simple antenna design facilitates the transformation of a non-stretchable conductive electrotextile into a stretchable layer with a minimal impact on its electrical performance. The LHD is formed by creating parallel, overlapping longitudinal slots of a specific length and width, which divide the sheet into repeated linked sections capable of stretching compared to a plain sheet without such longitudinal slots. This way, an array of parallel columns of longitudinal slots is formed; this array helps to sustain deformation in a uniaxial direction, while also enhancing the sheet’s flexibility when handled. These design principles are followed in our research to convert a non-stretchable conductive textile fabric into a stretchable structure by incorporating the LHD. This innovative approach opens up possibilities for diverse implementations and a variety of antenna designs to obtain enhanced antenna stretchability. Figure 3 provides a conceptual overview of the developed conformable, flexible, and stretchable microstrip patch antenna through an LHD.

One significant advantage of using e-textiles with a lower surface resistivity is the reduced cost of integrating antennas into garments. Additionally, our proposed LHD-based stretchable antenna fabrication method offers minimal change in its electrical performance while providing stretchable characteristics. The distinguishing feature of our research from the existing stretchable antenna literature lies in the simpler and novel technique employed to create a fully textile stretchable antenna. This work can be further extended by exploring different design patterns to convert non-stretchable materials into stretchable ones.

## 3. Materials and Methods

The selection of both conductive and non-conductive/dielectric materials for fabricating a stretchable antenna was primarily based on the factor of stretchability. In the recent literature, a variety of dielectric materials commercially available, such as Ecoflex, Solaris, and PDMS, have been utilized to fabricate stretchable antennas [3,37,38,39,40]. These soft, transparent, flexible, and stretchable polymers offer improved wearability for wearable applications compared to conventional rigid dielectric materials like FR4, which are traditionally used in antenna fabrication.

### 3.1. Conductive Textile Materials

**Figure 4:**
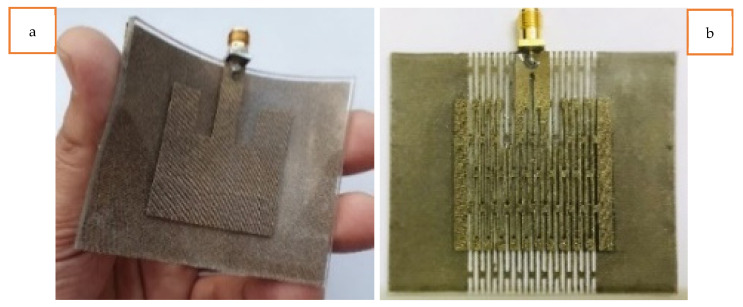
Shieldex^®^ Technik-tex P130 +B e-textile-based solid (**a**) and lattice hinge (**b**) stretchable patch antenna prototypes.

Among the various fabric geometries, knitted structures are the most suitable for fabricating stretchable textile antennas due to their superior stretchability compared to woven and non-woven structures. This research begins with the selection of the knitted conductive materials to be used in the stretchable antenna fabrication. Initially, a commercially available, two-way, stretchable, conductive, silver-coated, knitted electrotextile (Shieldex^®^ Technik-tex P130 +B by Statex Produktions, Bremen, Germany) was chosen because of its highly stretchable nature and lowest possible surface resistivity among the other commercially available knitted structures. This silver-plated knitted fabric is composed of 78% polyamide and 22% elastomer, with a coating of 99% pure silver. However, this fabric has a surface resistivity of 2 Ohm/square (Ω/sq) and a conductivity of 909 S/m, significantly lower than those of a standard copper metal sheet with a conductivity of 5.96 × 10^7^ S/m. Both a basic patch antenna prototype (without the LHD) and an LHD patch antenna prototype were simulated and fabricated to observe their behavior upon stretching. While both antenna prototypes exhibited stretchability, the stretchability of the LHD antenna prototype was particularly better when experienced by hand. The fabricated antenna prototypes are depicted in Figure 4. However, when the electric performances of these fabricated antenna prototypes were measured, as indicated by the S11 parameter, they were unsatisfactory, as the antenna did not achieve the −10 dB mark during resonance.

To fabricate a stretchable antenna with improved electrical performance, another conductive textile fabric, Shieldex^®^ Kiel +30 from Statex Produktions, Germany, was utilized. This fabric is copper-plated and non-woven in structure, but non-stretchable. However, the selection of Shieldex^®^ Kiel +30 only supports this research by offering a significantly lower surface resistivity of 0.02 Ω/sq and a high conductivity of 108.895 × 10^3^ S/m compared to the stretchable Shieldex^®^ Technik-tex P130 +B e-textile. Its surface resistivity is approximately 100 times lower compared to that of the Shieldex^®^ Technik-tex P130 +B fabric.

As the selected Shieldex^®^ Kiel +30 conductive fabric is non-stretchable, the possibilities for converting it into a stretchable material were examined. Here, the technique of converting a non-stretchable fabric into a stretchable fabric by implementing the LHD becomes relevant and adapted. Due to this innovative design approach to stretchable antenna fabrication, we finally settled on a non-stretchable copper-coated polyamide e-textile (Shieldex^®^ Kiel +30) with better electrical conductivity and transformed it into an LHD for use in the stretchable antenna. A higher electrical conductivity is essential for any conductive material used in antenna fabrication, as it minimizes electrical losses and contributes to the improved EM characteristics of the antenna. Table 1 tabulates all the related properties of both the Shieldex^®^ Technik-tex P130 +B and Shieldex^®^ Kiel +30 conductive fabrics.

### 3.2. Dielectric Substrate and Its Characterization

Among a variety of dielectric materials commercially available, as mentioned above, PDMS was selected. The choice of PDMS as the dielectric antenna substrate was made as it is a transparent, stretchable, and hyperplastic polymer. It offers a higher viscosity of 3500 centipoise, which strengthens the interfacial bonding between the dielectric substrate and the e-textiles. A 2 mm thick layer of the PDMS substrate (SYLGARD™ 184 Silicone Elastomer, Dow^®^, Washington, DC, USA) was prepared in a custom-made mold for fabricating the stretchable textile antenna. The PDMS was composed of two liquid components: the base and the curing agent, which were mixed in specific proportions (10:1). When the two components were thoroughly mixed, the mixture cured into a flexible and stretchable elastomer. The 10:1 mix ratio allowed for better control over the hardness and modulus, enabling improved stretchability and flexibility. With a higher Young’s modulus of 1.5 MPa, PDMS demonstrated a better conformation with human skin for on-body applications and could withstand high deformations.

The resonance frequency method was adopted to characterize the dielectric PDMS substrate [4,41]. The dielectric constant (E_r_) and loss tangent (tan δ) values needed to be known to simulate the final antenna prototype. To do so, an iterative process was employed, which involved changing the E_r_ value and observing the corresponding change in the resonance frequency of the antenna at a 2.45 GHz operating frequency using CST Studio Suite 2019, an Electromagnetic and Multiphysics simulation software. During the antenna simulation, both the properties of the selected conductive and dielectric PDMS materials were defined in the CST simulator to characterize the dielectric PDMS substrate. A simple rectangular microstrip patch antenna was simulated using the specific dimensions (length and width) of the 2 mm thick PDMS dielectric substrate, with arbitrary values of E_r_ and tan δ at the 2.45 GHz operating frequency. Subsequently, the antenna was physically fabricated. To compare the simulated results with the experimental results, the resonance frequency was measured using a Vector Network Analyzer (VNA). The difference between the resonance frequencies of the simulated and experimental antennas was initially observed. The E_r_ value in the CST simulator was then adjusted accordingly to match the simulated and experimental resonance frequencies. Once the resonance frequencies coincided with the new E_r_ value, it was considered to be the actual value for the PDMS substrate. The presence of substrate losses was reflected in a broader and less pronounced resonance peak in the return loss graph, with the depth of the peak indicating the level of the substrate losses. Similarly, the value of tan δ was manipulated to match the depth of both the simulated and experimental resonance frequency peaks. In this way, the dielectric PDMS substrate was characterized. The measured characteristics of the stretchable PDMS dielectric substrate at a frequency of 2.45 GHz are listed in Table 2.

## 4. Materials and Methods

### 4.1. Antenna Design and Simulation

**Figure 5:**
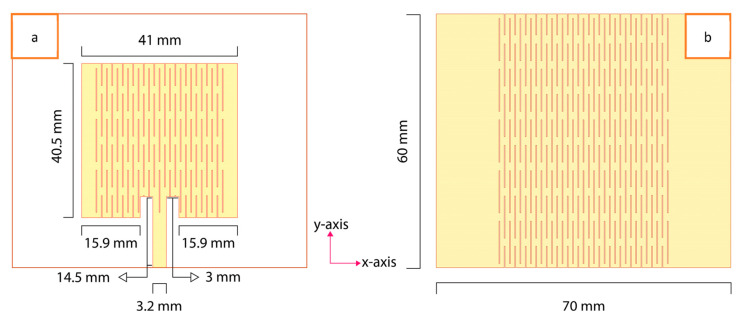
The front view (**a**) and the back view (**b**) of a simulated LHP stretchable antenna.

The microstrip patch antenna topology was chosen for its numerous advantages in wearable radio frequency applications. Planar patch antennas offer simplicity in design, lightweight construction, and easy integration into garments [42,43]. When fabricated using soft, flexible, and stretchable materials, such antennas become highly suitable for ergonomic use in wearable applications. Additionally, the planar patch antenna design minimizes the dissipation of EM radiation in human body tissues during wearable usage, thanks to the presence of a conductive ground plane. The microstrip patch antenna topology, depicted in Figure 3, consists of a dielectric substrate sandwiched between a radiating conductive patch and a conductive ground plane. This antenna topology is well-suited for intrinsic strain-sensing applications, where uniaxial strain can be easily applied to the antenna during wearability, as reported previously. The strain-sensing factor is detected by monitoring the shift in the resonance frequency caused by external strain. The magnitude of the frequency shift can be used to quantify the deformation. Figure 5a provides a schematic view of a simulated microstrip patch antenna design transformed into a stretchable antenna through the patterning of the conductive patch and ground plane in an LHD, as depicted from the front view, while Figure 5b shows the back view.

In wearable applications, the dimensions of the antenna play a crucial role in determining its operating resonance frequency. For the design of the LHD-based patch antenna, the length and width of the patch were calculated using the transmission line model. The physical dimensions of the antenna, particularly the patch length, were adjusted to achieve resonance at the desired frequency of 2.45 GHz during simulation.

The antenna design and simulation were conducted at an operating frequency of 2.45 GHz using the CST Studio Suite. The overall dimensions of the proposed LHP stretchable antenna in this research work are listed in Table 3. These dimensions were precisely cut using an automatic laser cutter, ensuring accurate fabrication.

### 4.2. Antenna Fabrication

**Figure 6:**
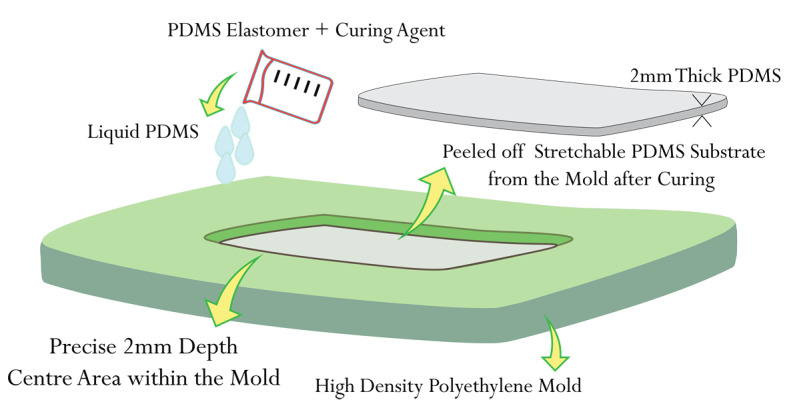
Schematic view of the PDMS substrate preparation process using a mold of high-density polyethylene.

A mold was designed using AutoDesk Fusion, a modeling software, to shape the liquid PDMS polymer. The mold was created from high-density polyethylene with precise dimensions of 80 × 70 × 2 mm^3^ (width × length × depth) using a Computer Numeric Control (CNC) machine. This mold was utilized to pour the liquid PDMS polymer, allowing it to take on the accurate shape specified above, as depicted in Figure 6. PDMS is a flowable liquid with excellent dielectric properties. It can be cured at various temperature ranges, from room temperature to 150 °C, and the curing time can range from 48 h to 35 min.

**Figure 7:**
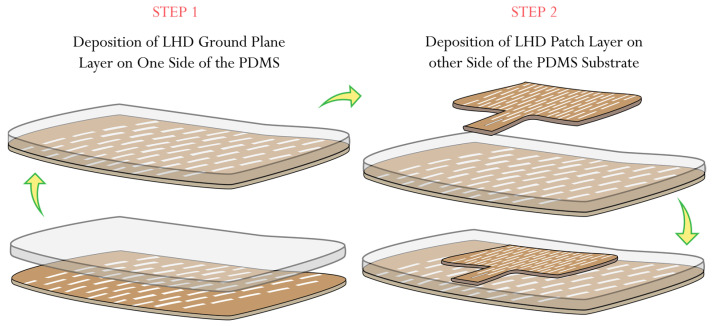
Shieldex^®^ Kiel +30-based LHP stretchable patch antenna.

The antenna fabrication process involved several steps. Initially, samples of the LHD for both the patch and the ground plane were cut from the Shieldex^®^ Kiel +30 e-textile fabric using a laser cutter. Next, the liquid PDMS polymer was prepared and placed in a desiccator to remove any air bubbles. Once prepared, the PDMS was poured into a pre-prepared mold with specific dimensions and placed in a curing chamber set at a temperature of 60 °C. A few minutes into the curing process, the surface of the liquid PDMS polymer started to solidify, becoming sticky. At this stage, the pre-cut sample of the conductive ground plane was carefully placed onto the partially cured sticky surface of the PDMS polymer and left to continue curing. The PDMS polymer typically took around 50 min to fully cure at 60 °C. Once fully cured, the PDMS polymer sample, with the conductive ground plane layer adhered on one side, was peeled off from the mold. Finally, a thin layer of fresh liquid PDMS polymer was applied to the other side of the fully cured PDMS polymer, and the patch layer was deposited, leveled, and left to cure once again at 60 °C. Through these process steps, the fabrication of both solid patch antenna samples and LHP patch antenna samples was carried out. Figure 7 provides a schematic view of the fabrication process for the LHP stretchable antenna samples, respectively. The porous nature of Shieldex^®^ Kiel +30 e-textile fabric facilitated better bonding with the PDMS polymer.

During the fabrication process, special attention was given to aligning the LHD links in the patch with the links in the ground plane. This alignment ensured that uniform stretchability was achieved throughout the fabricated antenna.

Considering the limitations of the chosen conductive e-textiles and the fabrication method involving laser cutting, it was determined that the clearance between adjacent links in the LHD should not be less than 2.5 mm on the materials chosen for our research work. The laser cutting of the selected e-textile fabric in an LHD provided flexibility but reduced its strength when the clearance was decreased to below 2.5 mm. On the other hand, reducing the clearance between adjacent links significantly increased the flexibility and stretchability of the e-textile, albeit at the expense of its strength. The length of the lattice hinge link played a critical role in the stretchability of the antenna, with a longer link length resulting in better stretchability. During the simulation, a trade-off was observed between the length of the link and the electrical performance of the antenna at the chosen resonance frequency. The width of the link should be minimized to maximize the available area for inserting more links into the patch and ground plane. Increasing the number of links in the conductive layers of the antenna enhanced its stretchability.

**Figure 8:**
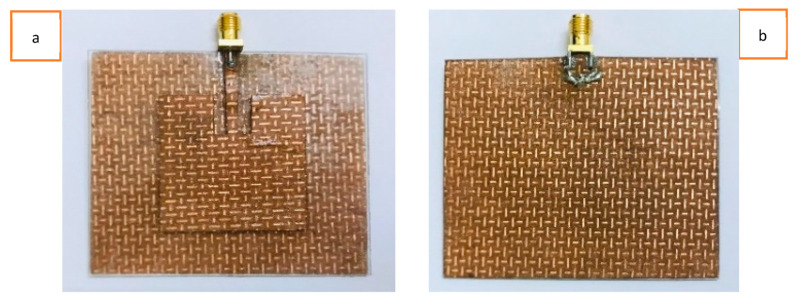
(**a**) Front view and (**b**) back view of Shieldex^®^ Kiel +30 e-textile-fabric-based solid antenna.

**Figure 9:**
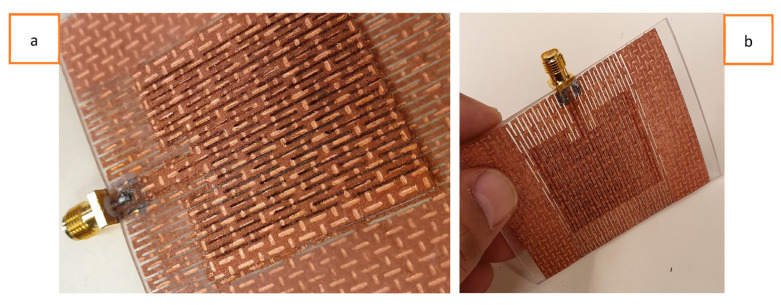
(**a**) Front view and (**b**) back view of Shieldex^®^ Kiel +30 e-textile-fabric-based LHP antenna.

Furthermore, rough antenna samples were fabricated with varying thicknesses of the PDMS substrate, ranging from 1.5 mm to 3 mm. It was found that thinner substrates provided better overall stretchability but reduced the strength of the antenna when stretched, and vice versa. Therefore, a 2 mm thick PDMS substrate was finally chosen, as it sustained a reasonably good deformation during stretching. Front and back views of the fabricated solid patch antenna using the Shieldex^®^ Kiel +30 e-textile are depicted in Figure 8a and Figure 8b, respectively. Similarly, front and back views of the fabricated LHP stretchable patch antenna using the Shieldex^®^ Kiel +30 e-textile fabric are shown in Figure 9a, and Figure 9b, respectively. Previous studies have also highlighted the trade-off between mechanical stretchability and EM performance in stretchable and flexible antennas, which aligns with our research. In summary, the overall mechanical stretchability of the antenna depended on the morphological parameters of the LHD, including the link width, link length, and clearance between adjacent links.

Based on the observation, the experimentally fabricated solid patch antenna using the Shieldex^®^ Kiel +30 e-textile fabric did not exhibit any stretchability, despite the stretchable nature of the PDMS substrate. However, in contrast, the experimentally fabricated LHP patch antenna using the Shieldex^®^ Kiel +30 e-textile offered some stretchability. Additionally, the antenna showed improved flexibility when manually handled. Each antenna prototype was soldered with an SMA connector with a 50 Ω characteristic impedance to provide power to the antenna through the microstrip feed line. It should be noted that the microstrip feed line in the LHP antenna was kept solid, without lattice hinge links, to ensure a robust connection to the SMA connector and enable a uniform flow of the input power.

## 5. Experimental Electromagnetic Characterization

**Figure 10:**
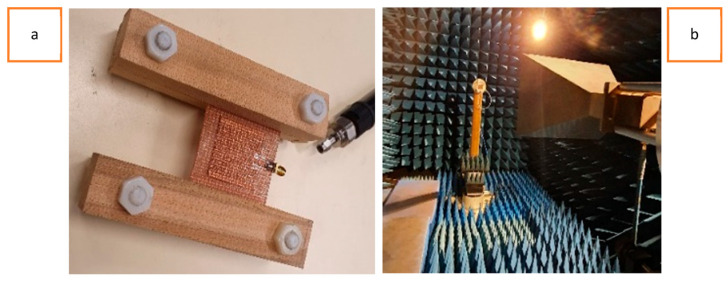
(**a**) Shows a custom-made setup to measure the reflection coefficient of the fabricated antenna and (**b**) depicts the measurement setup for radiation pattern measurements in an anechoic chamber.

The experimental characterization of the fabricated antenna prototype involved the use of a custom-made setup to evaluate its EM performance under uniaxial stretching. The EM performance of the antenna was assessed by measuring its S11 parameter at varying strain ranges. The S11 parameter represents the reflection coefficient of the antenna, indicating how well it can radiate power at a specific operating frequency. The power that was not reflected at the antenna’s interface to the transmission line was either radiated or resistively dissipated. However, due to the good conductivity of the materials used, the dissipation was assumed to be very low, which will be confirmed by the radiation pattern measurements further in this paper. By measuring S11 during the stretching process, the antenna’s performance under different strain conditions could be analyzed and evaluated. Figure 10a illustrates the custom-made setup used for the experimental characterization of the fabricated antenna prototype to measure its reflection coefficient. The setup involved the use of rectangular wooden blocks to clamp the antenna, with two blocks being placed on each side of the antenna in the widthwise direction. The blocks were bolted together using bolts made of high-density polyethylene material. Since the wooden blocks only touched the antenna’s ground plane and not its radiating patch, the influence of the wooden parts was very limited. Actually, during real-time measurements, the verification measurements showed no difference. The RF characterization of the antenna was performed using an Agilent E5071C Vector Network Analyzer (VNA) to measure the scattering parameters and observe the effect of applied strain on the resonance frequency.

The radiation patterns of the fabricated antenna prototype were measured in an anechoic chamber, as depicted in Figure 10b. The anechoic chamber provided a controlled environment free from reflections and external interference, allowing for accurate measurements of the antenna’s radiation pattern.

To measure the S11 of the antenna at different strain ranges, uniform uniaxial tension was applied using laser-cut wooden block spacings. These block spacings represented the amount of applied strain and were placed between the main wooden clamps of the custom-made setup. By adjusting the spacing, different elongation ranges were achieved, allowing for the measurement of S11 at varying strain levels. To validate the experimental results, the antenna prototype was also simulated at varying strain levels using the CST studio suite. The simulation results were compared with the experimental measurements to assess the correlation between both the simulated and experimental results. This comprehensive characterization process helped to understand the antenna’s behavior under strain and validate the effectiveness of the proposed design and fabrication methods.

## 6. Results and Discussion

**Figure 11:**
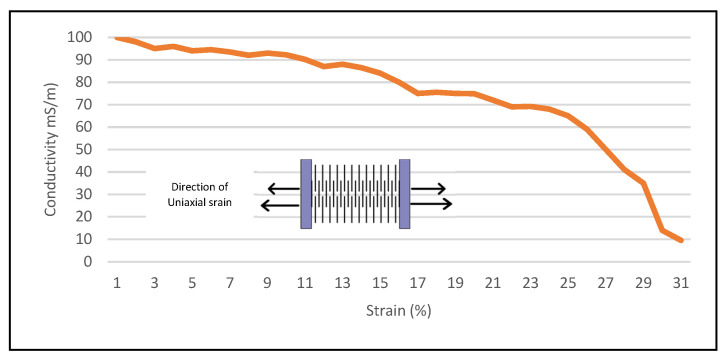
Illustration of the change in the conductivity of an LHP Shieldex^®^ Kiel +30-based stretchable patch antenna at different values of uniaxial tensile strain. The inset image shows the schematic view of the electromechanical test setup of the LHD with wooden clamps from both sides.

To see the effect of a change in the electrical properties (i.e., conductivity) of the LHP stretchable patch antenna upon uniaxial stretching, the same custom-made tensile setup, as mentioned in Figure 11, was used, involving a digital multimeter. Figure 11 indicates that, as the strain gradually and uniformly increased up to 25%, the end-to-end conductance of the patch decreased. At around a 30% strain, the conductivity reached approximately one-fifth of its starting value at a 0% strain. At strain levels beyond 30%, the antenna experienced failure observed as a decrease in conductance at multiple lattice hinge links in both the patch and the ground plane. Note that the DC values measured with the multimeter only provide a rough indication, as resistivity at higher frequencies is seriously influenced by skin and proximity effects.

**Figure 12:**
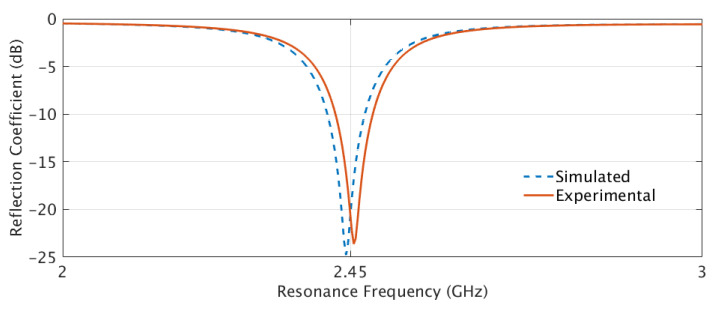
The simulated and experimental resonance frequency of Shieldex^®^ Kiel +30-based solid patch antenna at 2.45 GHz.

**Figure 13:**
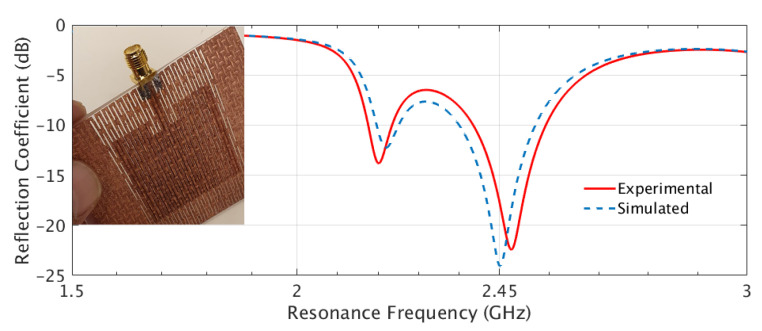
The simulated and experimental resonance frequency curves of LHP Shieldex^®^ Kiel +30-based stretchable textile patch antenna (inset image) at 2.45 GHz.

To measure the S11 of the fabricated antenna prototypes, a Vector Network Analyzer (VNA) was used. Before conducting the S11 measurements, a calibration process known as the short-open-load-through (SOLT) calibration was performed using a Keysight 85052D calibration kit. The SOLT calibration helped to establish a reference for accurate measurements by compensating for the inherent reflections and losses in the measurement setup. Figure 12 illustrates the resonance frequencies of the simulated and experimental results of the Shieldex^®^ Kiel +30-based solid patch antenna. Both the simulated and experimental results, in terms of S11, showed resonance at the 2.45 GHz operating frequency. This indicated a good agreement between the simulated and measured data. On the other hand, Figure 13 depicts the correlation between the simulated and experimental reflection characteristics of the LHP Shieldex^®^ Kiel +30-based stretchable patch antenna. The reflection coefficient measurements showed dual frequency peaks at 2.18 GHz and 2.45 GHz, both in the simulated and experimental data, when the LHD was implemented.

These results demonstrate the similarity between the simulated and experimental data, validating the accuracy of the simulation model and the successful fabrication of the stretchable patch antenna.

During the design and simulation of the LHP stretchable patch antenna, it was observed that the resonance frequency shifted towards the lower end when the LHD was incorporated into the patch and ground planes of the solid antenna. This shift in resonance frequency indicated the possibility of tuning the antenna to the desired operating frequency of 2.45 GHz by changing the solid antenna into the LHD antenna. To achieve the same frequency tuning in conventional antennas, the resonance frequency can be adjusted by changing the length of the patch or the thickness of the substrate. In our research, the focus was to design and simulate the LHP stretchable patch antenna at an operating frequency of 2.45 GHz. To achieve the desired resonance frequency, the length of the newly formed LHP stretchable patch antenna was adjusted compared to the patch length of the solid antenna. The incorporation of the LHD allowed for the transformation of a solid patch antenna into a stretchable and tunable LHP antenna suitable for wearable applications.

**Figure 14:**
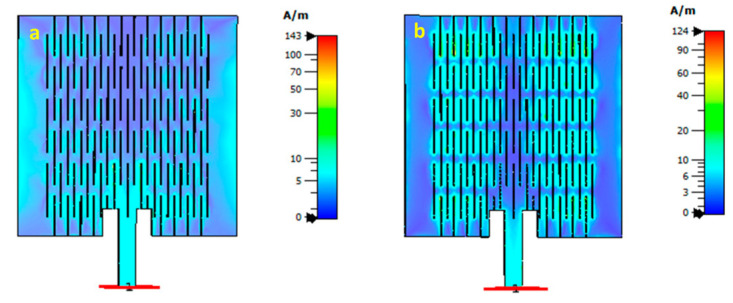
Effect of the surface current distribution at (**a**) 2.18 GHz resonance frequency peak, and (**b**) at 2.45 GHz resonance frequency peak for the LHD-based stretchable antenna, respectively.

The simulations and measurements revealed two resonant frequencies, 2.18 GHz and 2.45 GHz for the LHD-based stretchable patch antenna. The current distribution on the LHD-based stretchable patch antenna is illustrated in Figure 14a,b, for the 2.18 and 2.45 GHz resonance frequencies, respectively.

It can be observed from Figure 14a that there was an effect along the outer edges of the structure at the 2.18 GHz resonance frequency. The current density was largest in the bottom section, near the feedline. At this frequency, the resonance mode was similar to a regular patch antenna, with the original resonance being shifted downwards from 2.45 to 2.19 GHz due to the presence of the LHP. Interestingly, as visualized in Figure 14b for the current distribution at 2.45 GHz, it can be noticed that the current now flowed meandering around the lattice-hinge pattern and followed a zig-zag path along the lengths of the hinges throughout the lattice-hinge structure. A set of meandering currents were seen across the length of the patch, all contributing to the radiation, with a current minimum in the center of the patch. This was a new resonance mode that could only occur due to the presence of the lattice hinge structure.

**Figure 15:**
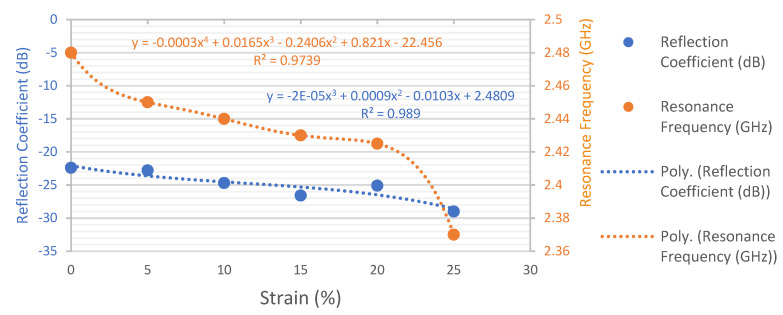
Change in the resonance frequency as a function of tensile strain up to 25% of fabricated LHP Shieldex^®^ Kiel +30-based stretchable textile patch antenna (inset image).

The effect of applied strain on the change in the resonance frequency and reflection coefficient values for the fabricated LHP stretchable patch antenna at different levels of elongation is illustrated in Figure 15. The antenna exhibited a maximum elongation of up to 25% before experiencing failure. It was observed that, upon a gradual increase in applied strain, the resonance frequency of the LHD stretchable antenna slightly shifted towards lower frequencies while experiencing a minor deviation in its reflection coefficient values at the same time. While discussing both the reflection coefficient and resonance frequency trends from the data mentioned in the graph, a linear trend was observed in the change in the resonance frequency from 2.48 to 2.41 GHz as the strain increased up to 20%. As the strain was further increased up to 25%, the resonance frequency trend of the LHD stretchable antenna observed a rapid change and started to show a non-linear trend and, at last, the antenna experienced a failure when strain was applied beyond 25%. On the other side, a linear trend in the change in the reflection coefficient values was observed upon a gradual increase in the applied strain up to 25%. Throughout the 25% elongation range, the reflection coefficient remained below −10 dB, indicating a good antenna performance. The change in resonance frequency before antenna failure at break suggested that the fabricated LHP stretchable patch antenna has the potential to function as a strain sensor with up to 25% elongation. This additional functionality enhances the versatility of the LHP stretchable antenna.

**Figure 16:**
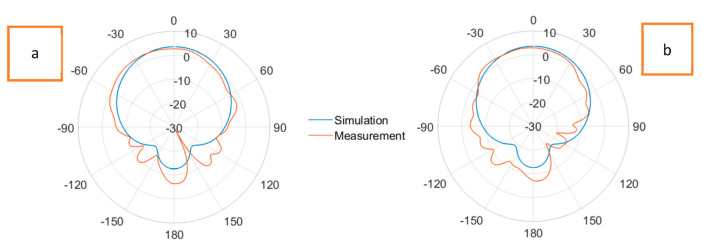
Radiation patterns at E-plane (**a**) and H-plane (**b**) of Shieldex^®^ Kiel +30-based solid patch antenna at 2.45 GHz.

**Figure 17:**
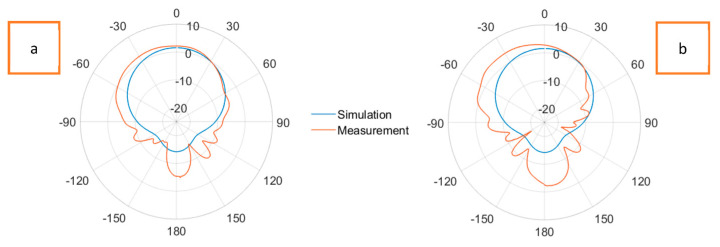
Radiation patterns at E-plane (**a**) and H-plane (**b**) of Shieldex^®^ Kiel +30-based LHP stretchable patch antenna at 2.45 GHz.

Figure 16 displays the radiation patterns of the Shieldex^®^ Kiel +30-based solid patch antenna at a 2.45 GHz resonance frequency in the (a) E-plane and (b) H-plane. The experimentally measured realized gains were 2.95 dBi and 2.98 dBi in the E-plane and H-plane, respectively. The illustrations shown in Figure 17 are the radiation patterns of the Shieldex^®^ Kiel +30-based LHP stretchable antenna at a 2.45 GHz resonance frequency in the (a) E-plane and (b) H-plane. The experimentally measured gains were 2.21 dBi and 2.34 dBi in the E-plane and H-plane, respectively.

In the radiation patterns of the Shieldex^®^ Kiel +30-based LHP stretchable antenna, a slightly higher level of back radiation can be observed in both the E-plane and H-plane compared to the Shieldex^®^ Kiel +30-based solid antenna. This is attributed to the presence of lattice hinge links in the ground plane, which reduced its overall conductive area and led to increased back radiation. However, the differences in the gains between the solid and LHP antennas were minimal, indicating that the stretchable nature of the LHP antenna did not significantly affect its radiation performance.

The Shieldex^®^ Kiel +30-based solid patch antenna did not exhibit stretchability despite the stretchy nature of the PDMS substrate. However, it did provide a slightly higher realized gain compared to the LHP stretchable antenna. The solid antenna remains flexible and can still be used effectively in wearable applications. On the other hand, the Shieldex^®^ Kiel +30-based LHP stretchable patch antenna compensated for this with enhanced flexibility and could stretch up to 25% in addition. The LHD significantly improved the overall flexibility and stretchability of the fabricated antenna, making it suitable for wearable applications where conformability and stretchability are desired.

## 7. Outcomes

In summary, our research aimed to design, fabricate, and characterize a multifunctional stretchable patch antenna operating at a resonance frequency of 2.45 GHz. The antenna was intended to serve as both a communication device and a strain sensor. Two antenna prototypes were fabricated using different conductive materials: Shieldex^®^ Technik-tex P130 +B (a stretchable knitted material) and Shieldex^®^ Kiel +30 (a nonwoven non-stretchable material) in search of better stretchability and electrical performance. The former antenna exhibited enhanced stretchability but had lower electrical performance. On the other hand, the latter antenna had a better electrical performance but limited stretchability. To address this trade-off, the LHD was incorporated into the non-stretchable antenna, improving its overall stretchability without significantly compromising its electrical performance.

The fabricated LHD stretchable patch antenna made from Shieldex^®^ Kiel +30 demonstrated stretchability of up to 25% under uniaxial tensile strain before it went to rupture. The antenna showed a linear trend in its resonance frequency shift as the applied strain increased up to 20% but continued to resonate without reaching a point of rupture. This characteristic makes it suitable for use as a strain sensor, in addition to its communication capabilities. The realized antenna gains at the E-plane and H-plane were measured as 2.21 dBi and 2.34 dBi, respectively.

Overall, our research successfully developed a multifunctional stretchable patch antenna at a 2.45 GHz resonance frequency with strain-sensing capabilities, providing conformability, flexibility, and stretchability for wearable applications while maintaining satisfactory electrical performance.

## Figures and Tables

**Table 1:** The EM characteristics of conductive textiles.

* Data Given by the Manufacturer	Shieldex^®^ Technik-Tex P130 +B	Shieldex^®^ Kiel +30
Fabric Type *	Knitted (stretchable)	Non-woven (non-stretchable)
Surface Resistivity * (Ω/sq)	2	0.02
Thickness * (mm)	0.55 mm ± 15%	0.250 mm ± 0.12 mm
Weight * (g/m^2^)	132 ± 18%	100 ± 15%

**Table 2:** Characteristics of the PDMS dielectric substrate.

	Thickness (mm)	Dielectric Constant (E_r_)	Loss Tangent (tan δ)
PDMS Substrate	2	2.7	0.01

**Table 3:** Dimensions of the simulated LHP stretchable antenna.

Parameters	Dimensions (mm)
Patch width (*x*-axis)	41
Patch length (*y*-axis)	40.5
Substrate/ground plane width	70
Substrate/ground plane length	60
PDMS substrate thickness	2
Lattice hinge link clearance/width	0.2
Lattice hinge link length	10
Clearance between adjacent links	3

## Data Availability

All the relevant data presented in this study are contained within this article.
